# Myocarditis and Pericarditis Post-mRNA COVID-19 Vaccination: Insights from a Pharmacovigilance Perspective

**DOI:** 10.3390/jcm12154971

**Published:** 2023-07-28

**Authors:** Abdallah Alami, Paul J. Villeneuve, Patrick J. Farrell, Donald Mattison, Nawal Farhat, Nisrine Haddad, Kumanan Wilson, Christopher A. Gravel, James A. G. Crispo, Santiago Perez-Lloret, Daniel Krewski

**Affiliations:** 1 School of Mathematics and Statistics, Carleton University, Ottawa, ON K1S 5B6, Canadanfarh033@uottawa.ca (N.F.); 2 McLaughlin Centre for Population Health Risk Assessment, University of Ottawa, Ottawa, ON K1N 6N5, Canada; 3 Department of Neuroscience, Faculty of Science, Carleton University, Ottawa, ON K1S 5B6, Canada; 4 School of Epidemiology and Public Health, University of Ottawa, Ottawa, ON K1G 5Z3, Canada; 5 Risk Sciences International, Ottawa, ON K1P 5J6, Canada; 6 Arnold School of Public Health, University of South Carolina, Columbia, SC 29208, USA; 7 Department of Medicine, University of Ottawa, Ottawa, ON K1H 8M5, Canada; 8 Bruyère Research Institute, Ottawa, ON K1R 6M1, Canada; 9 Ottawa Hospital Research Institute, Ottawa, ON K1Y 4E9, Canada; 10 Department of Epidemiology, Biostatistics and Occupational Health, McGill University, Montreal, QC H3A 1Y7, Canada; 11 Department of Mathematics and Statistics, University of Ottawa, Ottawa, ON K1N 6N5, Canada; 12 Faculty of Pharmaceutical Sciences, University of British Columbia, Vancouver, BC V6T 1Z3, Canada; 13 Division of Human Sciences, NOSM University, Sudbury, ON P3E2C6, Canada; 14 Consejo Nacional de Investigaciones Científicas y Técnicas (CONICET), Buenos Aires C1033AAJ, Argentina; 15 Observatorio de Salud Pública, Pontificia Universidad Católica Argentina, Buenos Aires C1107AAZ, Argentina; 16 Department of Physiology, Faculty of Medicine, University of Buenos Aires, Buenos Aires C1121ABG, Argentina

**Keywords:** VAERS, myocarditis, pericarditis, pharmacovigilance, vaccine safety, signal detection

## Abstract

Concerns remain regarding the rare cardiovascular adverse events, myocarditis and pericarditis (myo/pericarditis), particularly in younger individuals following mRNA COVID-19 vaccination. Our study aimed to comprehensively assess potential safety signals related to these cardiac events following the primary and booster doses, with a specific focus on younger populations, including children as young as 6 months of age. Using the Vaccine Adverse Events Reporting System (VAERS), the United States national passive surveillance system, we conducted a retrospective pharmacovigilance study analyzing spontaneous reports of myo/pericarditis. We employed both frequentist and Bayesian methods and conducted subgroup analyses by age, sex, and vaccine dose. We observed a higher reporting rate of myo/pericarditis following the primary vaccine series, particularly in males and mainly after the second dose. However, booster doses demonstrated a lower number of reported cases, with no significant signals detected after the fourth or fifth doses. In children and young adults, we observed notable age and sex differences in the reporting of myo/pericarditis cases. Males in the 12–17 and 18–24-year-old age groups had the highest number of cases, with significant signals for both males and females after the second dose. We also identified an increased reporting for a spectrum of cardiovascular symptoms such as chest pain and dyspnea, which increased with age, and were reported more frequently than myo/pericarditis. The present study identified signals of myo/pericarditis and related cardiovascular symptoms after mRNA COVID-19 vaccination, especially among children and adolescents. These findings underline the importance for continued vaccine surveillance and the need for further studies to confirm these results and to determine their clinical implications in public health decision-making, especially for younger populations.

## 1. Introduction

The field of pharmacovigilance involves monitoring the safety of therapeutic products under real-world conditions following market authorization [1]. In the context of vaccines, it encompasses the detection, assessment, understanding, prevention, and communication of adverse events following immunization (AEFIs) [1]. Immunization, a fundamental pillar of public health, has demonstrated remarkable effectiveness in reducing the burden of infectious diseases [1]. Preserving public confidence in vaccine safety is crucial for sustaining acceptance and continued uptake.

In the current global landscape, where the race to vaccinate populations against the enduring COVID-19 pandemic persists, employing newly authorized formulations and age-specific booster recommendations, the need to maintain public trust in vaccine safety has become even more pronounced. The two-messenger ribonucleic acid (mRNA) COVID-19 vaccines BNT162b2 (Pfizer-BioNTech) and mRNA-1273 (Moderna) have been shown to be generally safe in post-marketing surveillance systems [2]. However, concerns have been raised about the potential risk of myocarditis and pericarditis. These are rare but potentially serious adverse events characterized by inflammation of the heart muscle and/or outer sac [3,4]. Symptoms can range from mild to severe and include dyspnea, chest pain, fatigue, heart palpitations, and syncope, with most cases recovering completely within days after medical care [4]. Although the etiology of this mRNA vaccine-associated adverse event is still not well understood, several biological mechanisms underlying these adverse events have been hypothesized, including the dysregulated hyperactivated immune response [5,6], spike protein–myocardial protein cross-reactivity [6,7], and hormone-mediated upregulation of the immune response [8].

Given these concerns regarding possible cardiac adverse events linked to mRNA COVID-19 vaccines, ongoing analyses of recent reports of such events are critical to the identification and verification of these AEFIs. Safety signal detection involves identifying higher-than-expected reporting levels of AEFIs in comparison with population background rates or through a disproportionality analysis [9,10]. These safety signals often emerge from passive surveillance systems and are later confirmed and further evaluated through active surveillance systems [11]. The timely identification of myocarditis safety signals linked to mRNA COVID-19 vaccines has been made possible by the convergence of several factors, including established surveillance systems and vigilance by healthcare professionals [10]. Early reports from Israel identified an elevated myocarditis risk among vaccine recipients following the second dose of mRNA vaccines, with young men having a higher susceptibility [12]. Subsequently, major pharmacovigilance surveillance systems across the globe, including the U.S. VAERS [4], the European Union Drug Regulating Authorities Pharmacovigilance (EudraVigilance) [13], the Canadian Adverse Events Following Immunization Surveillance System (CAEFISSS) [14], the French spontaneous reporting system (Base Nationale de Pharmacovigilance, BNPV) [15], the United Kingdom Yellow Card scheme [16], and the World Health Organization’s VigiBase (Uppsala Monitoring Centre) [17], validated and reinforced these findings.

Despite ongoing efforts, research gaps persist, particularly regarding younger age groups. Most published studies on pharmacovigilance passive surveillance systems rely on data collected in 2021 [10], which inadequately address the potential risks for children or the impact of exposure to additional booster doses in adults. Our study expands upon previous analyses to capture data from younger individuals, including children 5 to 11 years of age, approved for vaccination in November 2021, and those as young as 6 months old, approved in June 2022 in the U.S. The objective of this study was to conduct a comprehensive analysis of potential safety signals associated with myocarditis and pericarditis following the administration of the primary and up to three booster doses of mRNA COVID-19 vaccines across different age groups and considering potential sex differences This included an assessment of the risk linked to repeated exposure and an evaluation of the vaccine-specific risks (Moderna and Pfizer-BioNTech mRNA COVID-19 vaccines) [14,18,19]. To optimize the sensitivity of signal detection and minimize the occurrence of false signals, we utilized both the frequentist and Bayesian methods in our disproportionality analyses, which allowed us to robustly evaluate reports of myocarditis and pericarditis associated with mRNA vaccines. Subgroup analyses were undertaken to focus specifically on safety signals in younger populations, specifically those less than 25 years of age, as they have been reported to be among the most affected [20,21,22]. We sought to explore all reported cardiovascular AEFIs for all vaccines for individuals under 25 years of age, focusing on the most frequently reported cardiac AEFIs in these populations, including myo/pericarditis, as well as other specific cardiac adverse events of potential concern.

Through this comprehensive approach, we aim to enhance our understanding of the cardiac safety profile of mRNA COVID-19 vaccines, with age- and sex-specific evaluations of the risks of myo/pericarditis and other cardiac events, thereby informing public health decision-making and vaccine safety surveillance.

## 2. Materials and Methods

### 2.1. Data Source

Our study utilized retrospective observational data obtained from VAERS [23], which is jointly administered by the US Centers for Disease Control (CDC) and US Food and Drug Administration (FDA) for the post-marketing safety surveillance of US-licensed vaccines [24]. VAERS contains AEFI reports from 1990 to the present and serves as an early warning system for potential vaccine safety issues. Its primary objective is to detect early safety signals and generate hypotheses concerning possible AEFIs not previously identified. VAERS receives spontaneous and voluntary reports from various stakeholders, including vaccine manufacturers, vaccine recipients, healthcare providers, and military personnel.

We retrieved raw data from the VAERS website from December 2020 to July 2022. The data were downloaded in the form of comma-separated value (.CSV) files for each year, containing three datasets:

(1) VAERS DATA dataset: consists of basic demographic information, including VAERS ID, sex, age, vaccination date, allergies, and health history.
(2) VAERS Symptoms dataset: consists of VAERS ID-linked adverse event symptoms, which may include up to five symptoms listed as being related to the administration of a vaccine and experienced by the vaccinee.
(3) VAERS Vaccine dataset: contains VAERS ID-linked information about the administered vaccine, including the vaccine type, manufacturer, lot number, number of doses given, route of administration, and site of injection.

### 2.2. Data Selection and Processing

For the study period, we extracted a total of 909,261 unique reports from the analytic dataset. To ensure the data quality, we conducted data cleaning and deduplication by removing reports with the same VAERS ID, unknown vaccine types, and reports with multiple co-administered vaccines to avoid the impact of co-vaccination. This resulted in 900,737 unique reports for analysis.

VAERS utilizes the Medical Dictionary of Regulatory Activities (MedDRA) to assign preferred terms (PTs) for coding the signs and symptoms of reported adverse events [25]. For this analysis, MedDRA version 23.0 was employed to analyze VAERS data for reports containing the following predefined PTs: “myocarditis”, “pericarditis”, and “myopericarditis”. Although the identified reports using the MedDRA terms do not represent confirmed diagnoses, all retrieved reports were examined and adjudicated using the CDC’s case definition of probable or confirmed myocarditis and pericarditis [4]. Specifically, for this analysis, identified AEFI reports required reporting of the PTs “myocarditis”, “pericarditis”, and “myopericarditis” and at least one concerning clinical symptoms and one abnormal cardiac test results or imaging observations consistent with these conditions (see Supplementary Table S1 for the detailed list of PTs used for adjudication).

Considering the considerable overlap in signs and symptoms, pathology, and clinical manifestations of myocarditis and pericarditis, all identified cases related to these two conditions were pooled and recoded into a single condition termed ‘myo/pericarditis’ in our analysis. For surveillance purposes, this term is used to refer to myocarditis, pericarditis, or myopericarditis throughout the present analysis. We included reports of myo/pericarditis with missing information on the vaccine manufacturer, dose number, or sex in descriptive statistics but excluded them from the disproportionality analysis, which is stratified by dose number and sex. We used R Statistical Software (version 4.1.3; R Foundation for Statistical Computing, Vienna, Austria) for data integration, extraction, and filtering, as well as for descriptive and inferential statistical analyses.

### 2.3. Statistical Analysis

Disproportionality methods are widely used in pharmacovigilance to detect higher than expected reporting rates of adverse events in large spontaneous reporting databases, such as the VAERS database [11]. These methods compute signal scores to identify unusual degrees of disproportionality between observed and expected values for a specific product-adverse event pair [11]. To ensure comprehensive safety surveillance, different frequentist and Bayesian methods of disproportionality are employed internationally [26,27,28], recognizing that no single approach is ideal for all scenarios, and multiple methods can be utilized to minimize the possibility of overlooking potential signals and differentiate between signal and background noise [29,30,31].

One of the most commonly used frequentist methods for signal generation is the Proportional Reporting Ratio (PRR), which is more sensitive than other signal detection methods [32,33,34]. Nonetheless, the PRR method has certain limitations, particularly in scenarios where small random fluctuations can significantly influence the PRR score [35]. In our analysis of myo/pericarditis, we foresee such circumstances, which can potentially lead to high false-positive rates, a known limitation of the PRR method. Furthermore, dealing with a cell count of zero can be challenging, since it generates a PRR of zero, leading to an indeterminate variance for ln(PRR). To mitigate this issue, we adhered to the common practice of setting a minimum cell count threshold for the PRR calculations; specifically, cells with counts less than three were excluded, as they are unlikely to provide a reliable signal of an adverse reaction [36]. Bayesian techniques such as the Bayesian Confidence Propagation Neural Network (BCPNN) have been introduced to address some of the limitations of classical disproportionality scores like the PRR method [37]. Although shrinking the disproportionality score towards a null value makes this technique less sensitive than the PRR method, it can mitigate the impact of artificially low expected counts and stabilize the observed-to-expected ratios in the presence of low counts. When combined with credible intervals, Bayesian techniques can minimize the risk of flagging false signals. A detailed discussion of these signal detection methods was provided by Farrell et al. (2018) [38].

The present analysis makes use of both the PRR and BCPNN methods, taking into account their respective strengths and limitations. We made this decision based on the understanding that no single method is superior to others in relation to all performance measures [30,39], consistent with recommendations to use various methods when conducting pharmacovigilance surveillance studies [40]. For signal detection using the PRR method, we required a lower bound of the 95% confidence interval (PRR_025_) of 1 or higher and a minimum of N ≥ 3 observations. Likewise, signal detection with BCPNN involved estimating the information component (IC) using the method proposed by Noren et al. (2011) [37]; a signal was detected when the 2.5% quantile of the posterior distribution of the IC (IC_025_ hereafter) was greater than zero. All signal detection methods were computed using the PhViD package in R [41], and a signal was affirmed only when both methods yielded signals. Adopting this approach in signal triage maintains the sensitivity while reducing the emphasis on signals based on limited data, resulting in an improved overall accuracy in signal detection.

Our primary analysis involved a disproportionality analysis of myo/pericarditis AEFI reports associated with mRNA vaccines compared to all other vaccines, encompassing all age groups. This analysis was stratified by a mRNA vaccine dose series (primary 1st; 2nd; or booster doses: 3rd, 4th, and fifth doses). As a sensitivity analysis, we also considered the possibility of masking effects—a statistical phenomenon that can obscure true signals of disproportionate reporting due to enhancements in the reporting of other vaccines with the event of interest, which can be precipitated by external influences [42]. This could introduce a bias in signal detection, as, if the event under study (myo/pericarditis) has a substantial reporting rate with other vaccines due to factors unrelated to the vaccine event risk, it could artificially inflate the background reporting rate, thereby reducing the sensitivity of the signal detection process [43]. Masking is especially likely in the context of novel mRNA COVID-19 vaccines and myo/pericarditis due to factors such as differences in media attention towards the two different mRNA vaccines, a smaller pharmacovigilance database with limited diversity, the rarity of the event, the novelty of these vaccines, and early-stage surveillance [44]. Public reporting of myo/pericarditis associated with both mRNA vaccines has presented inconsistent findings [14], with some studies suggesting a higher risk with Moderna compared to Pfizer-BioNTech [14,19] and others suggesting a greater risk with Pfizer-BioNTech [45,46,47], while others found no significant difference in risk [48]. These differences may be influenced by ascertainment bias and differences in the timing of the rollout of the two mRNA vaccines [14]. For instance, the Pfizer-BioNTech vaccine, which was more widely administered than the Moderna vaccine in the U.S. and was the first COVID-19 vaccine to receive FDA approval for use on adolescents, gained notoriety around the time when the myo/pericarditis case reports emerged, attracting the attention of the media, public, and healthcare providers alike. This could introduce bias when comparing the disproportionality of reporting myo/pericarditis of Moderna with that of Pfizer-BioNTech because of the different intensities of the media coverage of the two vaccines during the initial stages of deployment [14]. To explore the potential impact of this form of reporting bias, we conducted separate sensitivity analyses in which we restricted one of the two mRNA vaccine manufacturers, Moderna and Pfizer-BioNTech, respectively. By adopting this strategy, we aimed to ensure that our results were robust against potential bias resulting from masking effects and to evaluate if the risk of myo/pericarditis differs between the two mRNA vaccines.

We also conducted a temporal analysis to evaluate the signal trends in myo/pericarditis AEFIs over time for both mRNA vaccines. This involved generating a time scan map to illustrate the variations in signal strength over the study period. The emergence of a signal would be marked by a shift from a negative to a positive IC_025_ for the mRNA vaccine-AEFI combination. An upward trend and a narrowing 95% CI in IC value over time would suggest a stable and strong signal between the vaccine and the AEFI.

Recognizing that individuals younger than 25 years of age have been reported to be among the most affected [5,49,50,51,52], further exploration of the disproportionality safety signals within this population was pursued. We applied an age group classification that included infants, toddlers, and preschoolers (0–4 years); middle childhood (5–11 years); adolescents and young teens (12–17 years); and young adults (18–24 years) [53,54]. To enhance our understanding of AEFIs reported within these age groups, we developed a heatmap to visualize the distribution of AEFI reports by MedDRA’s system organ class (SOC) and the different vaccine types. These visual tools were designed to identify potential patterns or clusters in the distribution of AEFI reports among those younger than 25 years. Owing to the impracticality of analyzing the data at the PT level of the MedDRA hierarchy, which includes around 25,000 PT terms, we matched each reported PT term to a corresponding SOC level. Each PT must always be linked to at least one SOC, but it can be assigned to as many SOCs as applicable, resulting in the possibility of double counting. To avoid this limitation, we used a mapping approach published by Du et al. (2016) to assign each PT term a ‘primary SOC’ using the universally agreed-upon order of the SOC list (Table S6) [55].

Upon recognizing the emerging patterns of AEFI reporting across different organ systems illustrated in the heatmap, we conducted a more granular analysis of the cardiac SOC, narrowing our exploration on the reported AEFIs at the PT level within the cardiovascular system. This in-depth exploration was designed to provide a comprehensive evaluation of the full spectrum of cardiac adverse events reported by these age groups.

Lastly, following the apparent patterns of reported cardiovascular AEFIs with mRNA COVID-19 vaccines identified in the heatmaps, we performed a disproportionality analysis to further examine the potential signals between the five most frequently reported cardiac AEFIs following mRNA vaccination against COVID-19. We opted to focus this analysis on the primary dose series, as the booster doses were not authorized for all younger age groups at the time of the analysis. We also conducted a Time to Onset (TTO) analysis for each of the five most reported cardiac symptoms. Specifically, we calculated the mean and median times from the administration of the vaccine to the reported date of onset of each of these symptoms.

**Figure 1:**
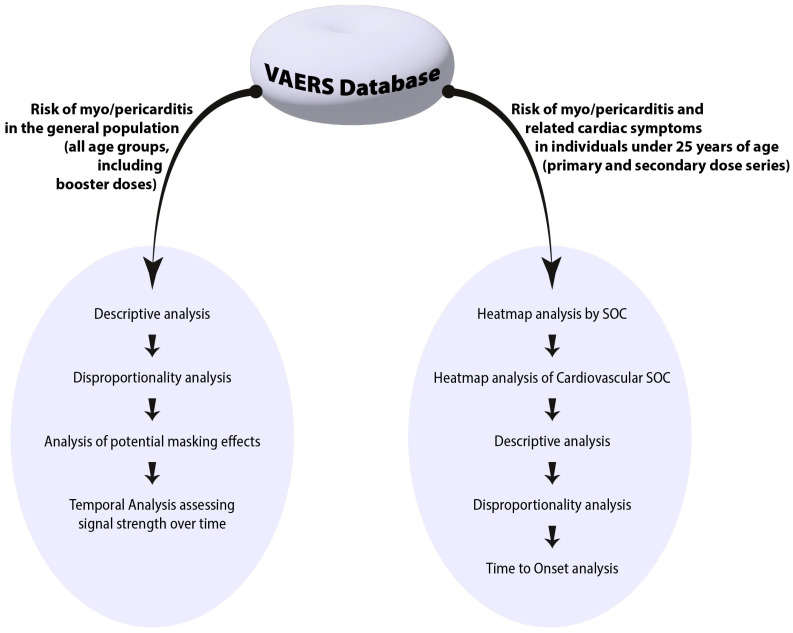
Flowchart illustrating the step-by-step analytical process used in evaluating data abstracted from the Vaccine Adverse Events Reporting System (VAERS).

The flowchart in Figure 1 provides a visual representation of our step-by-step analytical process, starting from the initial disproportionality analysis for all age groups and ending with the detailed examination of the five most reported cardiac adverse events of interest for the younger population under 25 years of age.

## 3. Results

### 3.1. Descriptive Analysis

Our analysis involved retrieving a total of 900,737 AEFI reports from VAERS between December 2020 and July 2022 covering a wide range of 73 different antigen and vaccine types, which are listed in the Supplementary Materials (Table S2). Our dataset included 4,084,226 unique report–AEFI combinations, recognizing the multidimensional nature of VAERS data, where a single report may contain multiple symptoms. An analysis of the VAERS data identified 5154 reports of myo/pericarditis during the study period. Of the COVID-19 vaccines considered, Pfizer/BioNTech had the highest number of reports at 3124 (60.6%), followed by Moderna with 1738 reports (33.7%) and Janssen with 204 reports (4%). The manufacturer name was missing in 24 reports (0.5%). Other vaccines had fewer reports of myo/pericarditis, including influenza vaccines (20 reports, 0.39%), varicella zoster vaccines (11 reports, 0.2%), smallpox vaccines (8 reports, 0.2%), meningococcal vaccines (7 reports, 0.1%), and pneumococcal vaccines (5 reports, 0.1%). The remaining vaccines, including human papillomavirus, anthrax, measles, mumps, rubella, varicella, rabies, hepatitis, Japanese encephalitis, typhoid, and yellow fever vaccines, had a total of 13 reports (0.3%) (Table S3). A single VAERS report of myo/pericarditis may have multiple outcomes. Hospitalization (*n* = 2966, 34.6%), emergency room or clinic visits (*n* = 2523, 29.4%), and doctor’s office visits (*n* = 1991, 23.2%) were the most frequently reported outcomes (Table S4).

**Figure 2:**
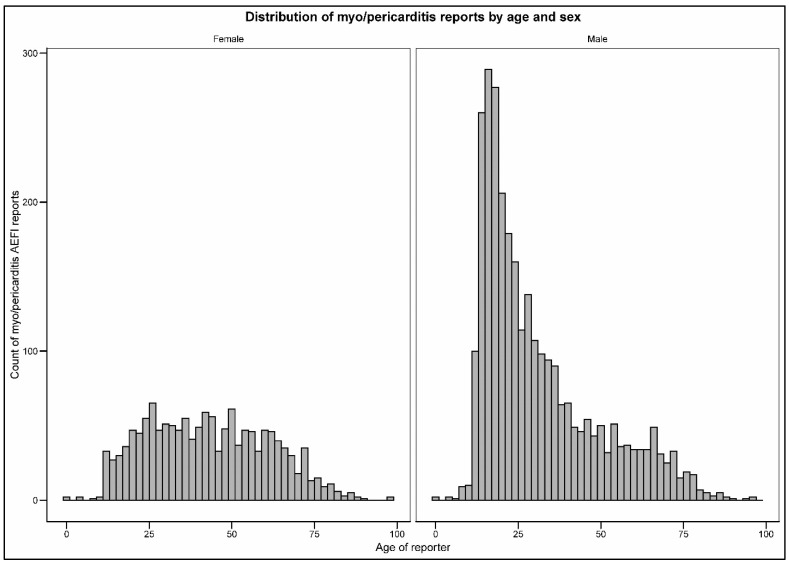
Age and sex distribution of individuals reporting myo/pericarditis adverse events in the Vaccine Adverse Event Reporting System following COVID-19 vaccination.

Approximately 37.6% of the total reported myo/pericarditis cases following vaccination were reported by individuals under the age of 25, with the highest percentage of reported cases in the 18–24 (20.6%) and 12–17 (16.8%)-year-old age groups. There were evident differences in the reporting trends between males and females for myo/pericarditis: males comprised the majority of reported cases (66.1%), and females accounted for 31.6% of the reports, with missing data on sex in the remaining (2.29%) reports. This difference was even more pronounced when analyzing the AEFI reports stratified by both age and sex. The distribution of reported cases in males exhibited negative skewness, indicating a notably higher number of reported cases occurring in younger age groups (Figure 2). Compared with other age groups, males aged 12–17 years (14.7%) and 18–24 years (17.1%) had the greatest number of reported myo/pericarditis cases. In contrast, females had a relatively low percentage of reported myo/pericarditis AEFIs across all age groups, with the highest percentage (5.6%) of cases occurring in the 31–40 and 41–50-year-old age groups combined (Table S5).

### 3.2. Signal Detection

We evaluated the disproportionality statistics produced by the two signal detection methodologies (PRR and BCPNN). The results of the analyses for the mRNA COVID-19 vaccines are shown in Table 1, according to vaccine type (all mRNA vaccines vs. other vaccines) and mRNA vaccine manufacturer (Pfizer-BioNTech or Moderna vs. other vaccines). The analyses were stratified by mRNA vaccine dose number (first or second primary series or third or fourth booster doses). There were only two myo/pericarditis cases reported following a fifth dose of a mRNA vaccine, which was insufficient for the signal analysis.

Our analysis identified that the majority of myo/pericarditis AEFIs reports with mRNA COVID-19 vaccines occurred after the primary series, with 3485 (87%) cases reported following the first or second doses. Notably, the second dose mRNA COVID-19 vaccines showed the strongest signals, with a disproportionality PRR score of 3.61 (95% CI 3.19–4.07) and an IC value of 0.38 (95% CI 0.31–0.43). The first dose also had a statistically significant disproportionality score of 1.58 (95% CI 1.39–1.79) and an IC value of 0.15 (95% CI 0.06–0.22). In contrast, we observed a lower number of reported myo/pericarditis cases for the booster doses, with only 495 (12%) reported following the third dose and 23 (0.6%) following the fourth dose. The PRR for the third dose was statistically significant, with a disproportionality score of 3.03 (95% CI 2.62–3.50) and an IC value of 0.81 (95% CI 0.66–0.92). However, no signal was detected following the fourth dose, with a PRR of 0.97 (95% CI 0.64–1.49) and an IC value of −0.04 (95% CI −0.74–0.45).

Our sensitivity analysis stratified by vaccine manufacturer, which accounted for the potential bias due to a possible masking effect, reinforced the validity of the initial signals for the mRNA COVID-19 vaccines and myo/pericarditis AEFIs identified in the aggregate analysis of both mRNA vaccines combined (Table 1). We observed a disproportionality signal for both Pfizer-BioNTech and Moderna vaccines, with a stronger signal observed for Pfizer-BioNTech than Moderna. Specifically, for the primary series, the Pfizer-BioNTech vaccine had a statistically significant PRR of 2.0 (95% CI 1.75–2.29) and IC value of 0.36 (95% CI 0.24–0.45) for dose 1 and a stronger signal with a PRR of 4.39 (95% CI 3.87–4.98) and IC value of 0.63 (95% CI 0.55–0.69) for dose 2. In comparison, the Moderna vaccine demonstrated a weaker signal with a PRR of 1.21 (95% CI 1.05–1.40) and IC value of 0.11 (95% CI −0.04–0.21) for dose 1 and a PRR of 2.62 (95% CI 2.29–3.0) and IC value of 0.56 (95% CI 0.43–0.64) for dose 2. For the booster doses, both vaccines had statistically significant PRRs and IC values for the third dose, but no significant signals were detected following the administration of the fourth or fifth doses of either vaccine.

### 3.3. Signal Trend Analysis over Time for Pfizer-BioNTech and Moderna

**Figure 3:**
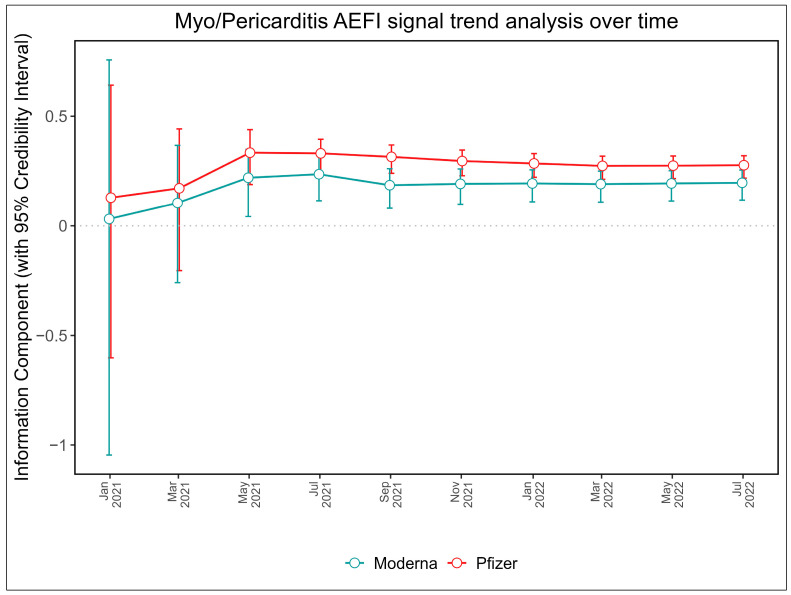
Trends in signal strength for myo/pericarditis following the administration of mRNA COVID-19 vaccines.

The analysis of signal trends for myo/pericarditis AEFIs over time revealed distinctive patterns for both mRNA vaccines. Our time scan map, as illustrated in Figure 3, showed a clear trajectory of the signal’s evolution. We observed that the value of the IC increased markedly with a steady upward trend early in March 2021 and that the lower 95% CI limit was above zero for both vaccines around May 2021 as the number of reports of mRNA-associated myo/pericarditis AEFI events increased.

### 3.4. Myo/Pericarditis after mRNA COVID-19 Vaccination in Younger Individuals

Our subgroup analyses focused on individuals under 25 years of age, a cohort that included children, teenagers, and young adults.

#### 3.4.1. Intensity of Report Counts by System Organ Class and Vaccine Type

**Figure 4:**
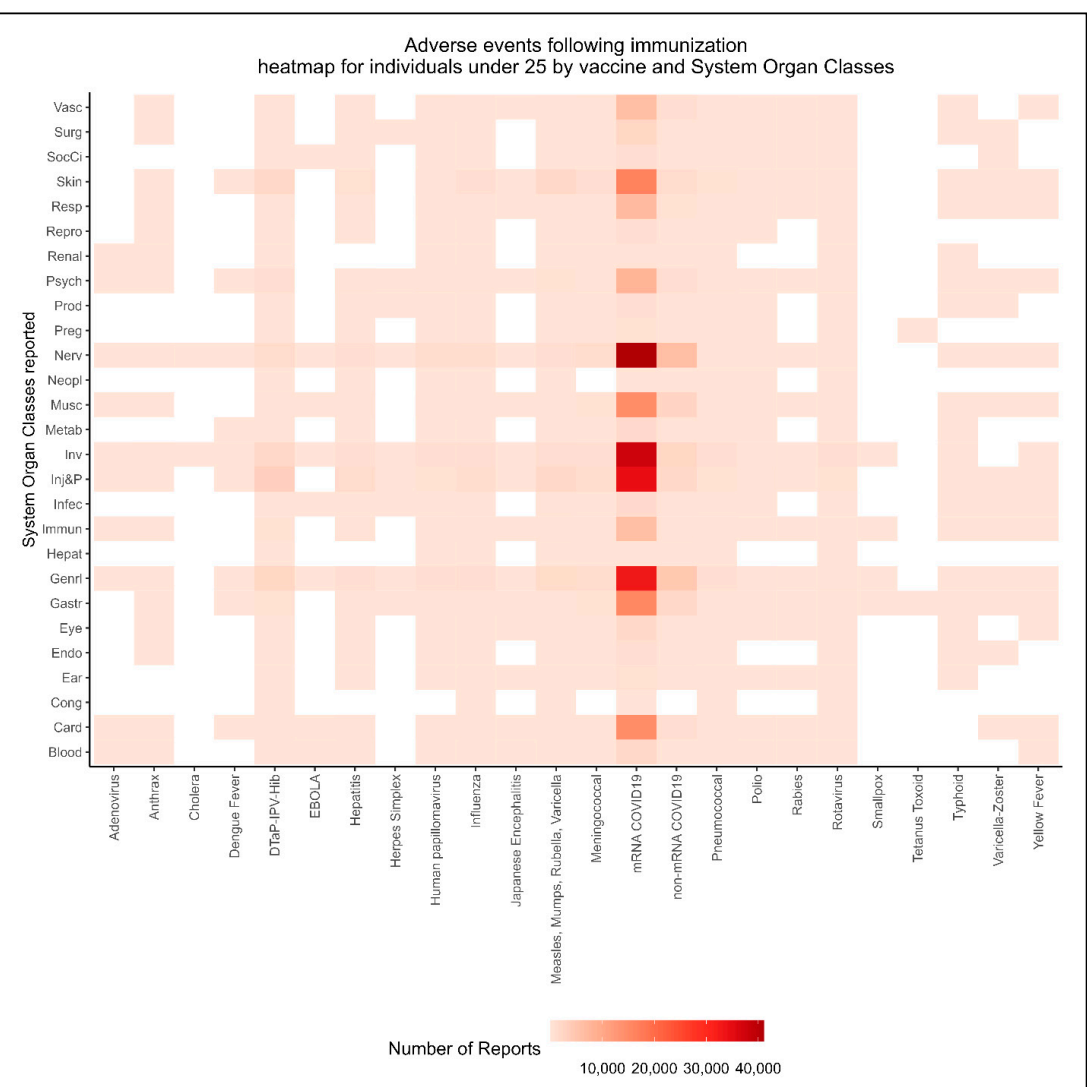
Heatmap analysis of Vaccine Adverse Events Reporting Patterns by system organ class in individuals under 25 years of age. The abbreviations used in the heatmap correspond to the following categories: Blood and lymphatic system disorders (Blood), Cardiac disorders (Card), Congenital, familial and genetic disorders (Cong), Ear and labyrinth disorders (Ear), Endocrine disorders (Endo), Eye disorders (Eye), Gastrointestinal disorders (Gastr), General disorders and administration site conditions (Genrl), Hepatobiliary disorders (Hepat), Immune system disorders (Immun), Infections and infestations (Infec), Injury, poisoning and procedural complications (Inj&P), Investigations (Inv), Metabolism and nutrition disorders (Metab), Musculoskeletal and connective tissue disorders (Musc), Neoplasms benign, malignant and unspecified (incl cysts and polyps) (Neopl), Nervous system disorders (Nerv), Pregnancy, puerperium and perinatal conditions (Preg), Product issues (Prod), Psychiatric disorders (Psych), Renal and urinary disorders (Renal), Reproductive system and breast disorders (Repro), Respiratory, thoracic and mediastinal disorders (Resp), Skin and subcutaneous tissue disorders (Skin), Social circumstances (SocCi), Surgical and medical procedures (Surg), and Vascular disorders (Vasc).

Within this young population, a total of 311,266 adverse event reports were identified in children, preteens, teens, and young adults ranging from 6 months to 24 years of age. The reported AEFIs, coded at the PT level of the MedDRA hierarchy, were matched with their primary SOC to facilitate a comprehensive visualization of the data. The resulting heatmap, illustrated in Figure 4, provided a visual summary of the patterns and distribution of reported AEFIs across the different organ systems. Lighter colors in the heatmap corresponded to smaller numbers of reports of this vaccine–SOC combination, while darker colors indicated higher numbers of reports. This heatmap showed the significant reporting of six main SOCs, including cardiovascular disorders, in conjunction with the receipt of a mRNA COVID-19 vaccine. This visualization provided a comprehensive overview of AEFI reporting patterns among individuals under 25 years of age and supported our hypothesis of a potential link between mRNA vaccines and the risk of cardiac complications.

**Figure 5:**
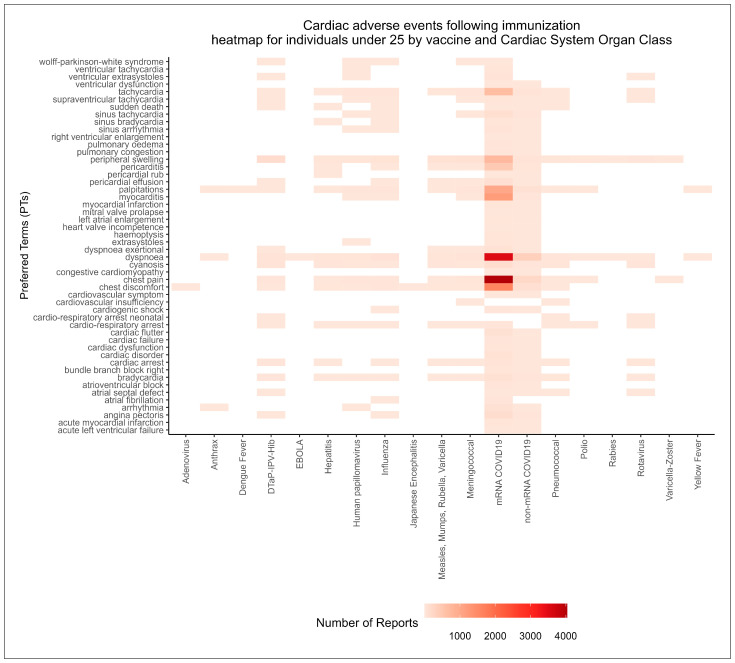
Heatmap of cardiovascular system Adverse Events Reporting Patterns for all vaccines in individuals under 25 years of age.

Our granular exploration of the cardiovascular SOC, portrayed in the heatmap in Figure 5, yielded important findings. This detailed visualization was constructed based on analyzing the entire cardiovascular SOC at the lower level of PTs of the MedDRA hierarchy across all vaccines for individuals under 25 years of age. The heatmap showed appreciable reporting of multiple cardiac symptoms following mRNA COVID-19 vaccination, including chest pain, dyspnea, tachycardia, and palpitations, in addition to myocarditis and pericarditis. While these symptoms can be interpreted as general symptoms of adverse cardiac events, they are also considered clinical manifestations of myo/pericarditis [56,57,58].

#### 3.4.2. Signal Detection for Cardiac AEFIs in Individuals Younger than 25 Years of Age

Building upon the preceding visual explorations, the disproportionality analyses provided further evaluation of the potential cardiovascular safety signals that were identified, prioritizing the five most frequently reported cardiac AEFIs associated with mRNA vaccines, as highlighted in the heatmaps. These included myo/pericarditis, as well as other related cardiac adverse events of concern, specifically chest pain, dyspnea, tachycardia, and palpitations (Table 2).

This analysis revealed notable age and sex differences in the reporting of myo/pericarditis cases. The youngest age groups, including children between 6 months and 4 years of age and between 5 and 11 years of age, had very few reported cases, with only 2 and 21 cases in the former and latter groups, respectively. However, in the 12–17 and 18–24-year-old age groups, the majority of cases occurred following the second dose, with males being more frequently affected than females. Specifically, in the 12–17-year-old age group, there were 408 reported cases, of which 363 were male and 45 were female, accounting for 88.7% and 11.0% of the cases, respectively. Similarly, in the 18–24-year-old age group, there were 433 reported cases, of which 367 were male and 66 were female, accounting for 84.8% and 15.3% of the cases, respectively. The analysis of the disproportionality scores revealed further insights: males showed significant signals following the second dose in middle childhood, young teens, and young adults, whereas the first dose was significant only for young male adults. Females exhibited a similar pattern, with significant disproportionality scores for the second doses only, with the strongest signal observed in young female teens following the second dose of the mRNA vaccine.

Further insights were obtained through the analysis of the other four cardiac AEFIs of interest: chest pain, dyspnea, palpitations, and tachycardia. An age-dependent increase in reporting these symptoms was evident in both sexes. Safety signals for chest pain and dyspnea were detected following the second dose of the mRNA vaccine in adolescents and young adults, with no significant differences observed between males and females. For palpitations, safety signals were detected with the first dose among young adult males. Conversely, young girls under the age of five showed safety signals for tachycardia following the first dose. In addition, our analysis of the TTO for each of these cardiac symptoms revealed further insights into their occurrence. For myo/pericarditis, the mean TTO was 5.9 days, with a median of 3 days. Similarly, palpitations had a mean TTO of 5.6 days and a median of 2 days. Chest pain and tachycardia exhibited similar patterns, demonstrating a mean TTO of approximately 6.9 and 7.2 days, respectively, and a median of 2 days. Lastly, dyspnea presented a mean TTO of 9.3 days and a median of 2 days. The short TTO observed across all symptoms supports a plausible association between the occurrence of these adverse events and COVID-19 vaccination.

## 4. Discussion

The potential risk of myo/pericarditis following mRNA COVID-19 vaccination continues to be a topic of ongoing discussion from a public health perspective. Our study contributes to the current body of evidence on the safety of COVID-19 vaccines by providing a comprehensive analysis of not only the reporting rates of myo/pericarditis following a booster or primary dose series but also explores other cardiovascular manifestations of this rare AEFI in children, adolescents, and young adults in a real-world population-based study. Using the VAERS dataset from December 2020 to July 2022, we identified 5154 reports of myo/pericarditis. Males had a notably higher number of reported cases (66.1%) than females (31.6%), with the majority of reported cases occurring in younger age groups. The disproportionality analyses, employing frequentist (PRR) and Bayesian (BCPNN) methods, generated safety signals for the primary series doses, with the second doses of the mRNA COVID-19 vaccines showing the strongest signal for myo/pericarditis. In contrast, a lower number of reported cases were observed for booster doses, and no significant signals were detected following the administration of the fourth or fifth doses of either mRNA vaccine. In contrast to previous studies that highlighted a higher risk following Moderna compared to the Pfizer-BioNTech COVID-19 vaccine [14,19,48], our stratified sensitivity analysis conducted to address the potential bias resulting from the masking effect identified safety signals for both mRNA vaccines following the second and third doses. Interestingly, we observed a slightly higher number of reports and a stronger signal with Pfizer-BioNTech compared to the Moderna mRNA COVID-19 vaccine.

We were also able to establish a timeline for the emergence and progression of these safety signals. Our time scan map points towards an initial detection of myo/pericarditis signals associated with both the Pfizer-BioNTech and Moderna vaccines as early as the first quarter of 2021. We subsequently observed a consolidation of this signal over the period from May 2021 to July 2022, which manifested as a narrowing of the 95% CI for the IC [59], indicative of a stable and strong signal for both mRNA COVID-19 vaccines and reports of myo/pericarditis AEFIs.

Our subgroup analyses examined the age- and sex-specific risks of myo/pericarditis following COVID-19 mRNA vaccines in the population most affected by this condition, comprised of individuals under 25 years of age. Recent studies have suggested an increased likelihood of developing myo/pericarditis in these age groups following mRNA COVID-19 vaccination [21,22,50]. However, these younger individuals also routinely receive several other vaccines, as per the U.S. national immunization schedule. Thus, our hypothesis proposed that this regular exposure to routine vaccines, coupled with the present increased awareness of vaccine-induced cardiac adverse events and extensive media attention regarding myo/pericarditis symptoms, could predispose this cohort to report myo/pericarditis symptoms with not only mRNA COVID-19 vaccines but also extend to other routine vaccines as well. With this perspective, our analysis of all reported cardiovascular AEFIs across all vaccines within this younger demographic revealed a significant trend of disproportionate reporting of cardiac symptoms post-mRNA COVID-19 vaccination. The symptoms were not confined to myo/pericarditis but also included chest pain, dyspnea, tachycardia, and palpitations. While these symptoms can be viewed as generalized adverse cardiac events, they are also recognized as clinical manifestations of myo/pericarditis. In many instances, these manifestations have been reported to be the main initial symptoms prompting cardiac magnetic resonance referrals following COVID-19 vaccine-associated acute myocarditis [60,61,62]. Furthermore, our analysis revealed notable distinctions in the reporting of myo/pericarditis cases based on age and sex, with males having the highest reported number of cases in the 12–17 and 18–24-year-old age groups. The signal was significant in both males and females following the second dose, yet the magnitude of this signal varied considerably between the two sexes. These findings align with several previous studies that have demonstrated an association between young individuals and a higher risk of myo/pericarditis through different types of epidemiological studies, including a spontaneous reporting system [16,17,63,64,65,66], case–control [67,68], self-controlled cases series [69,70,71,72], and cohort studies [19,48,73,74,75].

Myocarditis and pericarditis are not unique to mRNA COVID-19 vaccines, having been observed and reported with other vaccines like influenza and smallpox [76,77]. However, previous studies have indicated that vaccine-associated myo/pericarditis may be underestimated in passive surveillance systems [78,79,80,81]. Probable myocarditis in vaccine surveillance requires specific symptoms and abnormal cardiac test results, with confirmed cases further requiring histopathological or cardiac imaging confirmation, which can be difficult to achieve at the population scale [4]. Yet, infants and children under 12 years present with more general symptoms such as irritability, vomiting, poor feeding, tachypnea, and lethargy, complicating the diagnosis and reporting [4,82]. As a result, a passive vaccine safety surveillance system may fail to identify the signals of subclinical myocarditis in young age groups, as these populations are generally healthy, and myo/pericarditis can present in a relatively mild or asymptomatic form. This can lead to undiagnosed cases and underreporting [78,83], which is well known and described in the literature [84,85,86] and can contribute to the underestimation of this adverse event in the post-marketing surveillance phase of safety assessments. Although active surveillance can theoretically provide better case identification than passive surveillance [87,88], reporting still depends on medical suspicion and care seeking, which may be affected by the perception of a causal relationship with the vaccine, even among healthcare professional who underreport events that are not clearly related to a vaccination or to non-serious AEFIs [89].

Despite the inherent challenges, our study flagged a spectrum of cardiac-related symptoms that were reported more frequently than myo/pericarditis itself in younger age groups. This suggests that myo/pericarditis, particularly its non-severe forms, may be underdiagnosed under real-world conditions, leading to an underestimation of the actual risks within this demographic group [15]. Given the extensive media coverage and increased awareness of vaccine-induced cardiac adverse events, stimulated reporting could have contributed to the increased reporting of cardiac symptoms thought to be caused by COVID-19 vaccines. However, younger age groups who are regularly receiving routine vaccines may be predisposed to generate reports of myo/pericarditis symptoms not only with mRNA COVID-19 vaccines but also with other commonly used vaccines. Despite this, our study identified safety signals for chest pain and dyspnea following the second mRNA vaccine doses in adolescents and young adults, and these signals strongly align with the second dose pattern observed with reports of myo/pericarditis and mRNA vaccines, with no significant sex differences.

While the short-term clinical outcomes of COVID-19 vaccine-associated myocarditis are favorable, the ongoing monitoring and evaluation of the long-term effects of COVID-19 vaccine-associated myocarditis are crucial, especially in children and young people. Two recent studies examined the medium- and long-term outcomes of vaccine-induced myocarditis in adolescents and young adults. The medium-term study reported that most patients had achieved complete recovery, but a small percentage experienced chronic symptoms, including intermittent non-exertional chest pain and shortness of breath [90]. The long-term study found that, although most patients were considered recovered and cleared for physical activity, over half still exhibited cardiac abnormalities on follow-up cardiac MRIs, and around one-third were still prescribed daily medications related to myocarditis [91].

Despite the risk of myocarditis being significantly higher in individuals infected with SARS-CoV-2 compared to those who received the vaccine [92], there is no evidence to suggest that COVID-19 vaccines protect against myocarditis associated with SARS-CoV-2 infection [93]. These findings underscore the need for further research and surveillance to better understand the risks and benefits of COVID-19 vaccines, particularly in younger populations.

The strengths of our study include the use of VAERS as a comprehensive pharmacovigilance system, which has a broad national scope, potential for timely reporting, and the ability to identify rare events [94]. Additionally, our study provides a comprehensive analysis of the reporting rates of myo/pericarditis following the primary and booster doses of mRNA COVID-19 vaccines, as well as other cardiovascular manifestations of this rare adverse event in teens, adolescents, and young adults under real-world conditions, which adds new dimensions to our current understanding of this issue.

While our study provides valuable insights, it is important to consider the potential limitations. First, this study may be influenced by reporting biases, including underreporting (where not all vaccine-induced events are reported) [95], stimulated reporting (that could be triggered by media coverage and increased public awareness) [96], and other potential reporting biases inherent in spontaneous report databases [24,97,98]. Determining the extent to which these biases may have impacted our analysis remains challenging. Second, although we could not control for all relevant covariates, including prior COVID-19 infections, it remains uncertain whether individuals who have recovered from COVID-19 are at an increased risk of myo/pericarditis [83,99,100]. Third, it is important to note that higher disproportionality signal scores for mRNA vaccines may be influenced by the fact that these vaccines were the predominant vaccines used during the study period. Finally, given the invasive nature of a myocardial biopsy, its limited application for diagnosis may potentially constrain the accuracy in confirming reported cases in the VAERS system.

## 5. Conclusions

In summary, our study sheds light on the potential risk of myo/pericarditis following a mRNA COVID-19 vaccination in younger populations, highlighting the need for the ongoing monitoring and evaluation of this rare adverse event. The use of a disproportionality analysis utilizing both frequentist and Bayesian methods generated pharmacovigilance signals for the primary series doses, with the strongest signal observed following the administration of the second dose. Although a lower number of myo/pericarditis cases were reported for the booster doses, evidence of related cardiovascular symptoms post-mRNA immunization was detected in younger populations and warrants further study. While our study is subject to the potential limitations inherent in self-reporting databases, it underscores the importance of ongoing vaccine safety surveillance and the need for a better understanding of the risks and benefits of COVID-19 vaccines in younger populations. It is important to note that the reporting relationships flagged by the signal detection approaches do not imply causal associations and require confirmation through follow-up research, including analytic epidemiological studies, such as those based on electronic health records. Nevertheless, statistical signal detection provides a quick and efficient way to generate hypotheses and to explore potential risks within specific age groups, sexes, time frames, and product types, thereby providing early warnings of potential safety concerns.

## Figures and Tables

**Table 1:** Disproportionality analysis of myo/pericarditis reporting with mRNA vaccines compared to all other vaccines by mRNA vaccine type and dose series.

	Pfizer-BioNTech (Excluding Moderna Vaccine Reports)			Moderna (Excluding Pfizer-BioNTech Vaccine Reports)			All mRNA Combined		
**Vaccine Dose Number**	Cases	PRR (PRR_025_, PRR_975_)	IC (IC_025_, IC_975_)	Cases	PRR (PRR_025_, PRR_975_)	IC (IC_025_, IC_975_)	Cases	PRR (PRR_025_, PRR_975_)	IC (IC_025_, IC_975_)
**Dose 1**	736	2.0 (1.75, 2.29)	0.36 (0.24, 0.45)	524	1.21 (1.05, 1.40)	0.11 (−0.04, 0.21)	1260	1.58 (1.39, 1.79)	0.15 (0.06, 0.22)
**Dose 2**	1509	4.39 (3.87, 4.98)	0.63 (0.55, 0.69)	716	2.62 (2.29, 3.0)	0.56 (0.43, 0.64)	2225	3.61 (3.19, 4.07)	0.38 (0.31, 0.43)
**Dose 3**	308	3.47 (2.29, 4.07)	1.14 (0.95, 1.27)	187	2.05 (2.08, 3.0)	0.94 (0.69, 1.11)	495	3.03 (2.62, 3.50)	0.81 (0.66, 0.92)
**Dose 4**	13	1.07 (0.61, 1.86)	0.09 (−0.84, 0.74)	10	0.87 (0.46, 1.63)	−0.20 (−1.27, 0.53)	23	0.97 (0.64, 1.49)	−0.04 (−0.74, 0.45)
**Dose 5**	2	N/A	N/A	0	N/A	NR	2	N/A	N/A

PRR: Proportional Reporting Ratio; PRR_025_ and PRR_975_: Lower and Upper Bounds of the 95% Confidence Interval for PRR; IC: Information Component; IC_025_ and IC_975_: Lower and Upper Bounds of the 95% Confidence Interval for IC; N/A: Not available due to the small sample size. This was used in the table when N < 3 and no signal analysis was performed, following the best practices in pharmacovigilance and signal detection to prioritize the analysis when there were sufficient data. A signal was confirmed when the number of cases was greater than three, the lower bound of the PRR was greater than one, and the IC025 was greater than zero. Numbers in red indicated a signal was detected.

**Table 2:** Disproportionality analysis of mRNA COVID-19 vaccines and myo/pericarditis, chest pain, dyspnea, tachycardia, and palpitation in individuals under 25 years of age.

Adverse Event Following Immunization	mRNA Vaccine Dose Number	Age Groups:	Infants, Toddlers, and Preschoolers (6 Months to 4 Years)		Middle Childhood (5 to 11 Years)		Young Teens and Adolescents (12 to 17 Years)		Young Adults (18 to 24 Years)	
		Sex:	F	M	F	M	F	M	F	M
**Myo/pericarditis**	**1st dose**	Cases	0	0	2	7	21	110	41	141
		PRR (PRR_025_, PRR_975_)	N/A	N/A	N/A	4.8 (2.3, 10.0)	7.8 (1.1, 58.3)	4.1 (2.1, 8.1)	2.9 (1.1, 7.3)	3.3 (2.1, 5.2)
		IC (IC_025_, IC_975_)	N/A	N/A	N/A	0.8 (−0.6, 1.6)	0.4 (−0.3, 1)	0.3 (−0.1, 0.5)	0.3 (−0.3, 0.6)	0.4 (0.1, 0.6)
	**2nd dose**	Cases	1	1	1	11	45	363	66	367
		PRR (PRR_025_, PRR_975_)	N/A	N/A	N/A	12.2 (6.7, 21.9)	25.2 (3.5, 183.1)	15 (7.8, 29.1)	6.6 (2.7, 16.5)	11.7 (7.7, 17.8)
		IC (IC_025_, IC_975_)	N/A	N/A	N/A	1.1(0.1, 1.8)	0.6 (0.1, 1)	0.4 (0.3, 0.6)	0.5 (0.1, 0.8)	0.7 (0.5, 0.8)
**Chest pain**	**1st dose**	Cases	6	4	32	58	149	285	434	326
		PRR (PRR_025_, PRR_975_)	12.8 (3.2, 51.2)	5.7 (1.7, 18.9)	7 (2.1, 22.7)	3.6 (1.9, 6.9)	3.5 (2.1, 5.8)	4 (2.6, 6)	1.6 (1.3, 2)	1.9 (1.5, 2.4)
		IC (IC_025_, IC_975_)	2.3 (0.9, 3.2)	2.1 (0.3, 3.1)	0.6 (0, 1)	0.5 (0.1, 0.8)	0.3 (0, 0.5)	0.3 (0.1, 0.4)	0.1 (0, 0.3)	0.3 (0.1, 0.4)
	**2nd dose**	Cases	5	5	27	40	216	667	380	517
		PRR (PRR_025_, PRR_975_)	30 (7.2, 125.3)	31.6 (10.4, 95.9)	9.5 (2.9, 31.3)	4 (2.1, 7.8)	7.6 (4.6, 12.6)	10.4 (6.9, 15.6)	2 (1.6, 2.5)	4.1 (3.3, 5.1)
		IC (IC_025_, IC_975_)	3.6 (2, 4.6)	4.3 (2.7, 5.3)	0.9 (0.2, 1.3)	0.7 (0.2, 1.1)	0.5 (0.3, 0.7)	0.4 (0.3, 0.5)	0.3 (0.1, 0.4)	0.6 (0.4, 0.7)
**Dyspnea**	**1st dose**	Cases	27	15	41	39	249	184	754	324
		PRR (PRR_025_, PRR_975_)	7.2 (4.2, 12.5)	6.6 (3.5, 12.4)	0.9 (0.6, 1.5)	1.7 (0.9, 3)	1.8 (1.3, 2.4)	2.7 (1.7, 4.1)	1.3 (1.1, 1.5)	1.3 (1, 1.5)
		IC (IC_025_, IC_975_)	2 (1.3, 2.4)	2.2 (1.3, 2.8)	0 (−0.6, 0.3)	0.3 (−0.3, 0.6)	0.2 (0, 0.3)	0.2 (0, 0.4)	0.1 (0, 0.2)	0.1 (−0.1, 0.2)
	**2nd dose**	Cases	11	8	22	29	205	232	514	281
		PRR (PRR_025_, PRR_975_)	8.2 (4.1, 16.8)	15.5 (7.1,34)	0.8 (0.5, 1.4)	2 (1.1, 3.7)	2.2 (1.6, 3)	3.8 (2.5, 5.8)	1.3 (1.1, 1.5)	1.5 (1.2, 1.8)
		IC (IC_025_, IC_975_)	2.6 (1.6, 3.3)	3.6 (2.4, 4.4)	−0.2 (−0.9, 0.3)	0.4 (−0.2, 0.9)	0.3 (0.1, 0.5)	0.3 (0.1, 0.5)	0.1 (0, 0.2)	0.2 (0, 0.4)
**Palpitation**	**1st dose**	Cases	9	2	4	13	46	57	211	113
		PRR (PRR_025_, PRR_975_)	28.8 (6.2, 133.4)	N/A	0.2 (0.1, 0.7)	8.9 (1.2, 68)	1.1 (0.6, 1.9)	2.1 (1.1, 4.3)	1.3 (1, 1.7)	1.8 (1.2, 2.6)
		IC (IC_025_, IC_975_)	2.6 (1.5, 3.4)	N/A	−1.3 (−3, −0.2)	0.6 (−0.3, 1.3)	0 (−0.5, 0.4)	0.2 (−0.2, 0.5)	0.1 (−0.1, 0.3)	0.2 (−0.1, 0.5)
	**2nd dose**	Cases	3	0	7	6	50	70	145	94
		PRR (PRR_025_, PRR_975_)	27 (4.5, 161.3)	N/A	0.6 (0.2, 1.6)	6.6 (0.8, 55)	1.8 (1, 3.1)	2.9 (1.4, 5.8)	1.3 (0.9, 1.7)	2 (1.4, 3)
		IC (IC_025_, IC_975_)	3.5 (1.4, 4.7)	N/A	−0.4 (−1.7, 0.5)	0.9 (−0.6, 1.8)	0.2 (−0.2, 0.6)	0.3 (−0.1, 0.6)	0.1 (−0.2, 0.3)	0.3 (0, 0.6)
**Tachycardia**	**1st dose**	Cases	7	1	6	12	40	30	155	45
		PRR (PRR_025_, PRR_975_)	3.5 (1.4, 8.6)	N/A	3.9 (0.5, 32.5)	2.1 (0.7, 6.4)	0.8 (0.5, 1.4)	3.4 (1, 11)	1.7 (1.2, 2.5)	1.2 (0.7, 1.9)
		IC (IC_025_, IC_975_)	1.4 (0.1, 2.2)	N/A	0.5 (−0.9, 1.4)	0.3 (−0.6, 1)	−0.1 (−0.6, 0.3)	0.3 (−0.3, 0.7)	0.2 (−0.1, 0.4)	0.1 (−0.4, 0.4)
	**2nd dose**	Cases	2	1	6	5	36	27	114	46
		PRR (PRR_025_, PRR_975_)	N/A	N/A	6.3 (0.7, 52.6)	1.4 (0.4, 5.1)	1.1 (0.6, 1.9)	3.4 (1, 11.1)	1.8 (1.2, 2.7)	1.6 (1, 2.7)
		IC (IC_025_, IC_975_)	N/A	N/A	0.8 (−0.6, 1.7)	0.2 (−1.3, 1.2)	0.1 (−0.5, 0.4)	0.3 (−0.3, 0.8)	0.2 (−0.1, 0.5)	0.2 (−0.2, 0.6)

PRR: Proportional Reporting Ratio. PRR_025_ and PRR_975_: Lower and Upper Bounds of the 95% Confidence Interval for PRR. IC: Information Component. IC_025_ and IC_975_: Lower and Upper Bounds of the 95% Confidence Interval for IC. N/A: Not available due to the small sample size. This was used in the table when N < 3 and no signal analysis was performed following the best practices in pharmacovigilance and signal detection to prioritize the analysis when there were sufficient data. Numbers in red indicated a signal was detected.

## Data Availability

All data on the reports of myo/pericarditis submitted to the U.S. Food and Drug Administration’s Vaccine Adverse Events Reporting System are available for download from https://vaers.hhs.gov/data/datasets.html (accessed on 4 August 2022).

## Supplementary Materials

The following supporting information can be downloaded at: https://www.mdpi.com/article/10.3390/jcm12154971/s1: Table S1: MedDRA preferred terms used for the adjudication of AEFI reports with symptoms of myo/pericarditis. Table S2: Number of AEFI reports in VAERS by vaccine type and antigen. Table S3: Myo/pericarditis by vaccine type and manufacturer. Table S4: Reported outcomes for myo/pericarditis in the VAERS. Table S5: Sex-specific age distribution of reported myo/pericarditis adverse events following vaccination in VAERS data. Table S6: MedDRA terminology SOC list based on the internationally agreed order.
