# Supplementary material for: Myocarditis and Pericarditis Post-mRNA COVID-19 Vaccination: Insights from a Pharmacovigilance Perspective
Source: J Clin Med. 2023 Jul 28;12(15):4971. doi: 10.3390/jcm12154971 (PMC10419493; doi:10.3390/jcm12154971)
Supplement: Supplementary file 1 [file jcm-12-04971-s001.zip › jcm-2471377-supplementary.pdf]

Journal of
Clinical Medicine

MDPI

Supplementary Material

Myocarditis and Pericarditis Post mRNA COVID-19 Vaccination: Insights from a Pharmacovigilance Perspective

Abdallah Alami, Paul J. Villeneuve, Patrick J. Farrell, Donald Mattison, Nawal Farhat, Nisrine Haddad, Kumanan Wilson, Christopher A. Gravel, James A. G. Crispo, Santiago Perez-Lloret, Daniel Krewski

The supplementary material includes six tables providing additional details on the analyses and findings reported in this article. Table S1 lists the MedDRA preferred terms used for adjudication of AEFIS reports with symptoms of myocarditis and pericarditis according to the CDC's case definition for probable myocarditis. Table S2 presents the number of AEFIS reports in VAERS, categorized by vaccine type and antigen. Table S3 offers information on myo/pericarditis cases sorted by vaccine type and manufacturer. Table S3 shows reported outcomes for myo/pericarditis in the VAERS database. Table S4 highlights the sex-specific age distribution of reported myo/pericarditis adverse events following vaccination in VAERS data. Lastly, Table S5 contains the MedDRA terminology SOC list, arranged according to the internationally agreed order.

Table S1: MedDRA preferred terms used for adjudication of AEFIS reports with symptoms of myo/pericarditis

**Cardiac symptoms**		
Accelerated idioventricular rhythm	ECG signs of myocardial infarction	Right atrial volume increased
Arrhythmia	ECG signs of myocardial ischaemia	Right ventricular ejection fraction decreased
Arrhythmia supraventricular	ECG signs of ventricular hypertrophy	Right ventricular systolic pressure decreased
Atrial fibrillation	Echocardiogram abnormal	Right ventricular systolic pressure increased
Atrial flutter	Ejection fraction abnormal	Sinoatrial block
Atrial parasystole	Ejection fraction decreased	Sinus arrhythmia
Atrial tachycardia	Electrocardiogram abnormal	Sinus bradycardia
Atrioventricular block	Electrocardiogram ambulatory abnormal	Sinus tachycardia
Atrioventricular block complete	Electrocardiogram ST segment depression	Stress echocardiogram abnormal
Atrioventricular block first degree	Electrocardiogram ST segment elevation	Stroke volume decreased
Atrioventricular block second degree	Electrocardiogram ST-T change	Stroke volume increased
Atrioventricular conduction time shortened	Electrocardiogram ST-T segment abnormal	Supraventricular extrasystoles
Atrioventricular dissociation	ElectrocardiogramST-T segment depression	Supraventricular tachyarrhythmia
Atrioventricular node dispersion	Electrocardiogram ST-T segment elevation	Supraventricular tachycardia
Atrioventricular node dysfunction	Electrocardiogram T wave abnormal	Tachyarrhythmia
Biopsy heart abnormal	Exercise electrocardiogram abnormal	Tachycardia
Biopsy pericardium abnormal	Extrasystoles	Tachycardia paroxysmal
Bradyarrhythmia	Intracardiac pressure increased	Trifascicular block
Bradycardia	Left atrial volume abnormal	Troponin I increased
Bundle branch block	Left atrial volume decreased	Troponin increased
Cardiac fibrillation	Left ventricular end-diastolic pressure decreased	Troponin T increased

Cardiac flutter	Left ventricular end-diastolic pressure increased	Ventricular arrhythmia
Cardiac function test abnormal	Long QT syndrome	Ventricular asystole
Cardiac imaging procedure abnormal	Magnetic resonance imaging abnormal	Ventricular extrasystoles
Cardiac index abnormal	Myocardial hypoperfusion	Ventricular fibrillation
Cardiac index decreased	Myocardial oedema	Ventricular flutter
Cardiac index increased	Myocarditis	Ventricular parasystole
Cardiac monitoring abnormal	Myopericarditis	Ventricular pre-excitation
Cardiac output decreased	Palpitations	Ventricular tachyarrhythmia
Cardiac output increased	Parasystole	Ventricular tachycardia
Cardiac ventriculogram abnormal	Paroxysmal arrhythmia	
Cardiac ventriculogram left	Paroxysmal atrioventricular block	
Cardiac ventriculogram left abnormal	Pericardial effusion	
Cardiac ventriculogram right abnormal	Pericardial rub	
Cardiovascular function test abnormal	Pericarditis	
Central venous pressure abnormal	Right atrial pressure increased	
Conduction disorder	Right atrial volume abnormal	
Defect conduction intraventricular	Right atrial volume decreased	
	**Clinical symptoms**	
Chest discomfort		
Chest pain		
Dyspnoea		
Dyspnoea at rest		
Dyspnoea exertional		
Dyspnoea paroxysmal nocturnal		
Infantile spitting up*		
Infantile vomiting*		
Irregular breathing*		
Irritability*		
Lethargy*		
Neonatal tachypnoea*		
Nocturnal dyspnoea		
Orthopnoea		
Poor feeding infant*		
Syncope		
Tachypnoea*		

* infants and children <12 years of age may instead require at least 2 of these PT terms as clinical symptoms

Table S2. Number of AEFIs reported in VAERS by vaccine type and antigen.

Vaccine code	Vaccine description	N
**6VAX-F**	Diphtheria and tetanus toxoids and acellular pertussis adsorbed + inactivated poliovirus + hepatitis B + haemophilus B conjugate vaccine	9
**ADEN_4_7**	Adenovirus type 4 and 7 vaccine, live oral	64
**ANTH**	Anthrax vaccine	243
**BCG**	Bacillus Calmette-Guerin vaccine	20
**CHOL**	Cholera vaccine	42
**COVID19**	Coronavirus 2019 vaccine	1,216,221
**DF**	Dengue fever vaccine	64
**DT**	Diphtheria and tetanus toxoids, pediatric	41
**DTAP**	Diphtheria and tetanus toxoids and acellular pertussis vaccine	1,443
**DTAPH**	Diphtheria and tetanus toxoids and acellular pertussis vaccine +	3
**DTAPHEPBIP**	Diphtheria and tetanus toxoids and acellular pertussis vaccine + hepatitis B + inactivated poliovirus vaccine	841
**DTAPIPV**	Diphtheria and tetanus toxoids and acellular pertussis vaccine + inactivated	1,411
**DTAPIPVHIB**	Diphtheria and tetanus toxoids and acellular pertussis vaccine + inactivated poliovirus vaccine + haemophilus B conjugate vaccine	1,020
**DTOX**	Diphtheria toxoid	1
**DTP**	Diphtheria and tetanus toxoids and pertussis vaccine	24
**DTPHEP**	Diphtheria, tetanus, pertussis + hepatitis B	1
**DTPPVHBHPB**	Diphtheria and tetanus toxoids and acellular pertussis vaccine + inactivated poliovirus vaccine + haemophilus B conjugate vaccine + hepatitis B	137
**EBZR**	Ebola Zaire vaccine	65
**FLU(H1N1)**	Influenza (H1N1) monovalent	2
**FLU3**	Influenza virus vaccine, trivalent	263
**FLU4**	Influenza virus vaccine, quadrivalent	8,642
**FLUA3**	Influenza virus vaccine, trivalent, adjuvant	161
**FLUA4**	Influenza virus vaccine, quadrivalent, adjuvant	1,146
**FLUC3**	Influenza virus vaccine, trivalent, cell-culture-derived	108
**FLUC4**	Influenza virus vaccine, quadrivalent, cell-culture-derived	1,088
**FLUN3**	Influenza virus vaccine (nasal spray)	7
**FLUN4**	Influenza virus vaccine quadrivalent (nasal spray)	105
**FLUR3**	Influenza virus vaccine, trivalent, recombinant	6
**FLUR4**	Influenza virus vaccine, quadrivalent, recombinant	761
**FLUX**	Influenza virus vaccine, unknown manufacturer	3,580
**FLUX(H1N1)**	Influenza (H1N1) monovalent, unknown manufacturer	16
**H5N1**	Pandemic flu vaccine	1
**HEP**	Hepatitis B virus vaccine	2,590
**HEPA**	Hepatitis A	2,547
**HEPAB**	Hepatitis A + hepatitis B	416

**HEPATYP**	Inactivated hepatitis a + typhoid polysaccharide vaccine adsorbed	2
**HIBV**	Haemophilus B conjugate vaccine	1,633
**HPV4**	Human papillomavirus quadrivalent	653
**HPV9**	Human papillomavirus 9-valent	3,278
**HPVX**	Human papillomavirus (no brand name)	36
**IPV**	Poliovirus vaccine inactivated	737
**JEV1**	Japanese encephalitis virus vaccine, inactivated	41
**JEVX**	Japanese encephalitis virus vaccine (no brand name)	12
**MEN**	Meningococcal polysaccharide vaccine	93
**MENB**	Meningococcal group b vaccine, rDNA absorbed	1,487
**MMR**	Measles, mumps and rubella virus vaccine, live	2,539
**MMRV**	Measles, mumps, rubella and varicella vaccine live	2,261
**MNQ**	Meningococcal conjugate vaccine	2,449
**MNQHIB**	Meningococcal groups c and y + haemophilus B tetanus toxoid conjugate	1
**MU**	Mumps virus vaccine, live	1
**OPV**	Poliovirus vaccine trivalent, live, oral	3
**PER**	Pertussis vaccine	2
**PNC**	Pneumococcal 7-valent conjugate vaccine	54
**PNC10**	Pneumococcal 10-valent conjugate vaccine	2
**PNC13**	Pneumococcal 13-valent conjugate vaccine	2,174
**PNC15**	Pneumococcal 15-valent Conjugate Vaccine	17
**PNC20**	Pneumococcal 20-valent conjugate vaccine	379
**PPV**	Pneumococcal vaccine, polyvalent	3,070
**RAB**	Rabies virus vaccine	366
**RV1**	Rotavirus vaccine, live, oral	365
**RV5**	Rotavirus vaccine, live, oral, pentavalent	1,329
**RVX**	Rotavirus (no brand name)	37
**SMALL**	Smallpox vaccine	63
**SMALLMNK**	Smallpox + monkeypox vaccine	41
**TD**	Tetanus and diphtheria toxoids, adult	343
**TDAP**	Tetanus toxoid, reduced diphtheria toxoid and acellular pertussis vaccine, adsorbed	3,151
**TDAPIPV**	Tetanus, diphtheria and acellular pertussis, and inactivated polio virus	2
**TTOX**	Tetanus toxoid	51
**TYP**	Typhoid vaccine	300
**UNK**	Unknown	16,958
**VARCEL**	Varivax-varicella virus live	3,028
**VARZOS**	Varicella-zoster vaccine	24,164
**YF**	Yellow fever vaccine	193

Table S3. Myo/pericarditis by vaccine type and manufacturer

Vaccine manufacturer	Vaccine type	N
PFIZER\BIONTECH	COVID19	3,124
MODERNA	COVID19	1,738
JANSSEN	COVID19	204
UNKNOWN MANUFACTURER	COVID19	24
GLAXOSMITHKLINE BIOLOGICALS	Varicella-Zoster	8
SANOFI PASTEUR	Smallpox	7
SEQIRUS, INC.	Influenza	7
UNKNOWN MANUFACTURER	Influenza	7
NOVARTIS VACCINES AND DIAGNOSTICS	Meningococcal	6
MERCK & CO. INC.	Pneumococcal	5
GLAXOSMITHKLINE BIOLOGICALS	Influenza	3
MERCK & CO. INC.	Human papillomavirus	3
MERCK & CO. INC.	Measles, Mumps, Rubella, Varicella	2
MERCK & CO. INC.	Varicella-Zoster	2
SANOFI PASTEUR	Influenza	2
EMERGENT BIOSOLUTIONS	Anthrax	1
GLAXOSMITHKLINE BIOLOGICALS	Hepatitis	1
NOVARTIS VACCINES AND DIAGNOSTICS	Rabies	1
PFIZER\WYETH	Meningococcal	1
PROTEIN SCIENCES CORPORATION	Influenza	1
SANOFI PASTEUR	Yellow Fever	1
UNKNOWN MANUFACTURER	Anthrax	1
UNKNOWN MANUFACTURER	Japanese Encephalitis	1
UNKNOWN MANUFACTURER	Rabies	1
UNKNOWN MANUFACTURER	Smallpox	1
UNKNOWN MANUFACTURER	Typhoid	1
UNKNOWN MANUFACTURER	Varicella-Zoster	1

Table S4. Reported outcomes for myo/pericarditis in VAERS

Outcome	N
Hospitalized	2,966
Emergency room/department or urgent care visit	2,523
Doctor or other healthcare professional office	1,991
Life threatening	726
Disability	274
Death	84
Congenital anomaly or birth defect	10

Table S5. Sex-Specific Age Distribution of Reported Myo/Pericarditis Adverse Events Following Vaccination in VAERS Data

Sex	Age group	N	Percentage
**F**	6 months-4 years	2	0
**M**	6 months-4 years	2	0
**F**	5-11 years	5	0.1
**M**	5-11 years	22	0.5
**F**	12-17 years	90	2
**M**	12-17 years	649	14.7
**F**	18-24 years	154	3.5
**M**	18-24 years	754	17.1
**F**	25-30 years	161	3.7
**M**	25-30 years	377	8.6
**F**	31-40 years	246	5.6
**M**	31-40 years	431	9.8
**F**	41-50 years	246	5.6
**M**	41-50 years	247	5.6
**F**	51-60 years	226	5.1
**M**	51-60 years	207	4.7
**F**	61-70 years	184	4.2
**M**	61-70 years	170	3.9
**F**	71-80 years	84	1.9
**M**	71-80 years	98	2.2
**F**	81-90 years	22	0.5
**M**	81-90 years	19	0.4
**F**	91+ years	3	0.1
**M**	91+ years	4	0.1

Table S6. MedDRA terminology SOC list based on internationally agreed order.

**SOC**	**Rank**
Infections and infestations	1
Neoplasms benign, malignant and unspecified (incl cysts and polyps)	2
Blood and lymphatic system disorders	3
Immune system disorders	4
Endocrine disorders	5
Metabolism and nutrition disorders	6
Psychiatric disorders	7
Nervous system disorders	8
Eye disorders	9
Ear and labyrinth disorders	10
Cardiac disorders	11
Vascular disorders	12
Respiratory, thoracic and mediastinal disorders	13
Gastrointestinal disorders	14
Hepatobiliary disorders	15
Skin and subcutaneous tissue disorders	16
Musculoskeletal and connective tissue disorders	17
Renal and urinary disorders	18
Pregnancy, puerperium and perinatal conditions	19
Reproductive system and breast disorders	20
Congenital, familial and genetic disorders	21
General disorders and administration site conditions	22
Investigations	23
Injury, poisoning and procedural complications	24
Surgical and medical procedures	25
Social circumstances	26
Product issues	27
